# Asymptomatic and Symptomatic Patients With Non-severe Coronavirus Disease (COVID-19) Have Similar Clinical Features and Virological Courses: A Retrospective Single Center Study

**DOI:** 10.3389/fmicb.2020.01570

**Published:** 2020-06-26

**Authors:** Yanli Li, Jie Shi, Jianbo Xia, Jie Duan, Lijuan Chen, Xudong Yu, Weishun Lan, Quanfu Ma, Xufeng Wu, Yichong Yuan, Liyan Gong, Xinghai Yang, Han Gao, Chunchen Wu

**Affiliations:** ^1^Department of Gynecology, Maternal and Child Health Hospital of Hubei Province, Tongji Medical College, Huazhong University of Science and Technology, Wuhan, China; ^2^Department of Laboratory Medicine, Maternal and Child Health Hospital of Hubei Province, Tongji Medical College, Huazhong University of Science and Technology, Wuhan, China; ^3^Department of Pediatric Neurology, Maternal and Child Health Hospital of Hubei Province, Tongji Medical College, Huazhong University of Science and Technology, Wuhan, China; ^4^Department of Radiology, Maternal and Child Health Hospital of Hubei Province, Tongji Medical College, Huazhong University of Science and Technology, Wuhan, China; ^5^Department of Surgery, Maternal and Child Health Hospital of Hubei Province, Tongji Medical College, Huazhong University of Science and Technology, Wuhan, China

**Keywords:** COVID-19, asymptomatic, symptomatic, chest CT, SARS-CoV-2

## Abstract

The current outbreak of coronavirus disease 2019 (COVID-19) has been defined as a pandemic by the World Health Organization. We aimed to evaluate the clinical features and virological course of non-severe COVID-19 patients with or without symptoms who were admitted to a Chinese cabin hospital. In this retrospective single center study, we reviewed 252 laboratory-confirmed COVID-19 patients treated at one temporary cabin hospital in Wuhan, China. Demographic, clinical, serial chest computed tomography (CT), and serial viral test data were compared between asymptomatic and symptomatic patients. The association between clinical features and symptomatic status or patient referral status was analyzed. Among all 252 patients, 74 (29.4%) were asymptomatic and 138 (54.76%) had more than two family members who developed COVID-19. The probability for family clustering was similar between asymptomatic and symptomatic patients (59.70 vs. 61.64%, *P* = 0.79). Asymptomatic patients and symptomatic patients were equally likely to reach a virus-free state during their stay at the cabin hospital (93.15 vs. 86.44%, *P* = 0.13). The initial chest CT screening showed that 81 (32.1%) patients had no visible pneumonia, 52 (20.6%) had unilateral pneumonia, and 119 (47.2%) had bilateral pneumonia. Symptomatic patients had a higher chance to have bilateral pneumonia (*P* < 0.0001) and were less likely to show improvement on the follow-up CT scan (*P* = 0.0002). In total, 69 (27.4%) patients were referred to the designated hospital and only 23 (9.1%) patients were referred due to the progression of pneumonia. Non-severe COVID-19 patients can transmit the disease regardless of their symptomatic status. It is highly recommended that asymptomatic patients be identified and quarantined to eliminate the transmission of COVID-19.

## Introduction

The severe acute respiratory syndrome coronavirus 2 (SARS-CoV-2), which causes coronavirus disease 2019 (COVID-19), is currently spreading rapidly around the world (Zhou et al., 2020a). On March 11, COVID-19 was characterized as a pandemic by the World Health Organization (WHO) (WHO). It is now affecting more than 180 countries and territories worldwide (WHO, 2020a,b). During the early stages of the coronavirus outbreak in Wuhan, China, the medical resources of the regular healthcare system were quickly drained and infected patients with non-severe symptoms were suggested to self-quarantine at home, which led to severe cases of family-clustered transmission and fast spread among communities. To relieve the strain on regular hospitals and eliminate the spread of disease by asymptomatic and mildly symptomatic patients, 16 temporary cabin hospitals were built to accommodate coronavirus patients with non-severe symptoms. In China, the outbreak has been brought under control through city lockdowns and building cabin hospitals; however, asymptomatic infection raises concerns about the recurrence of the epidemic. We found that many patients in cabin hospitals were asymptomatic, and there were many cases of familial clustering. It is important to evaluate the virological course, clinical features, and outcomes of asymptomatic, and symptomatic patients with COVID-19 treated at cabin hospitals, and share our experience with other countries, and territories that are currently dealing with the infectious disease.

The clinical spectrum of COVID-19 can range from asymptomatic infection to mild upper respiratory tract illness to severe interstitial pneumonia with respiratory failure and even death (Chen N. et al., 2020; Huang et al., 2020; Wang et al., 2020b). It is estimated that non-severe patients with no symptoms or mild symptoms could represent ~30–60% of all infections (Mizumoto et al., 2020; Nishiura et al., 2020; Qiu, 2020; Wang et al., 2020a). Compared to severe cases, asymptomatic infection and mildly symptomatic infection often go unrecognized since the majority of affected individuals are not sick enough to seek medical help and cannot be identified by screening methods, such as temperature check. A few studies have shown that high viral loads can be detected in some patients with COVID-19 early in their illness, when their symptoms were mild (Woelfel et al., 2020; Zou et al., 2020). Moreover, another asymptomatic patient was found to shed a similar amount of virus as that shed by symptomatic patients (Zou et al., 2020). Therefore, asymptomatic infection may be highly contagious and potentially lead to viral spread. Some transmission models also suggested that a substantial number of undocumented infections leading to mild, limited, or no symptoms may facilitate the rapid dissemination of SARS-CoV-2 (Gostic et al., 2020; Li et al., 2020). However, at present, there is little information regarding the clinical features of asymptomatic and mildly symptomatic infection. Moreover, details of the chest computed tomography (CT) scans and virologic course in these patients have not yet been well-documented or described.

In this study, we retrospectively analyzed the clinical data of 252 asymptomatic or mildly symptomatic COVID-19 patients admitted to a temporary cabin hospital in Wuhan. We described the clinical course of symptoms, changes of imaging features, and viral shedding during hospitalization, and explored the probability of clinical worsening of asymptomatic, and mildly symptomatic patients. We aimed to compare clinical features and the virological courses between asymptomatic and symptomatic patients.

## Materials and Methods

### Study Design and Participants

**Figure 1:**
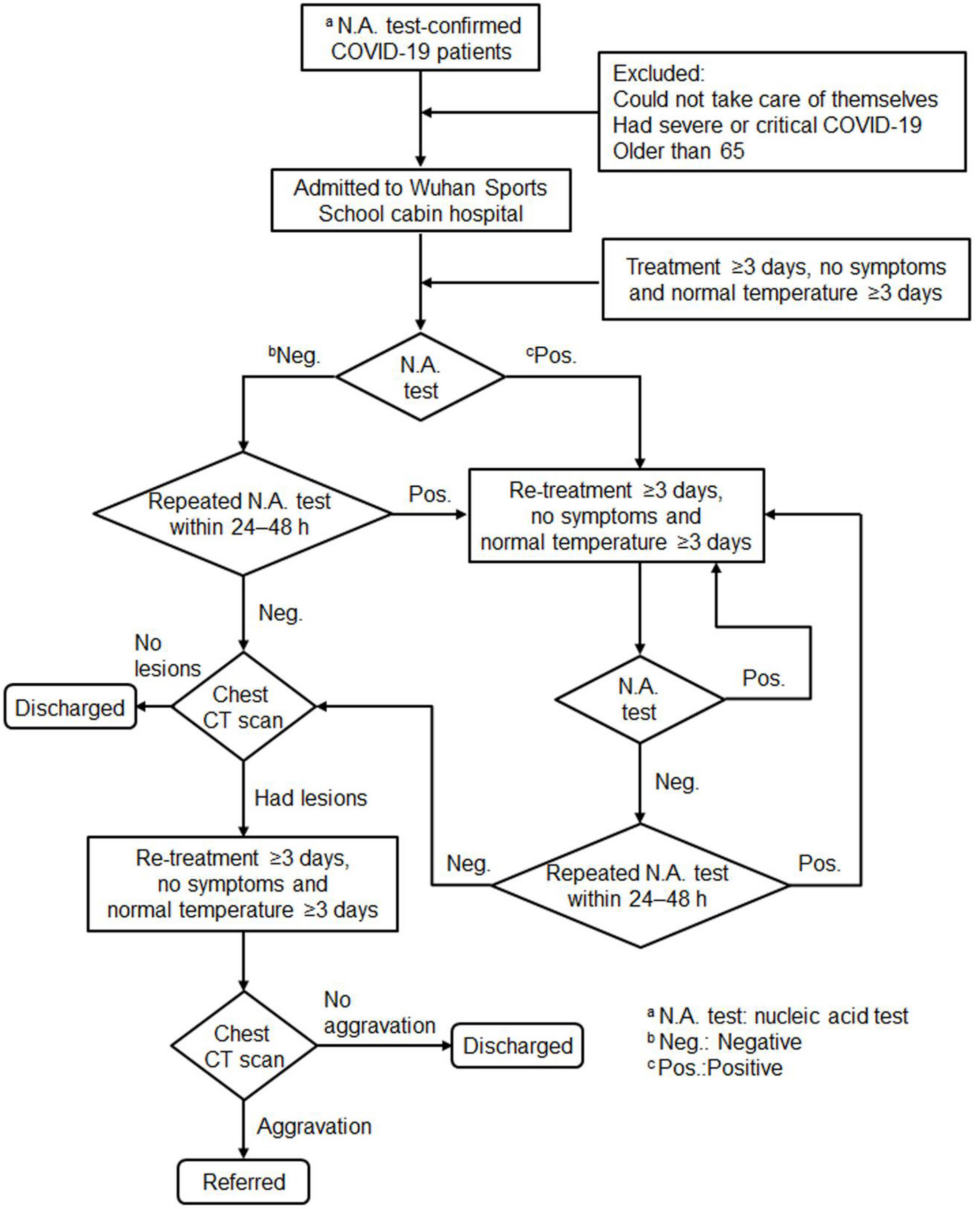
Flow diagram of patients enrolled in this study. Patients were admitted to the cabin hospital after being diagnosed with coronavirus disease 2019 (COVID-19) following laboratory confirmation of severe acute respiratory syndrome coronavirus 2 (SARS-CoV-2) using polymerase chain reaction (PCR) testing; excluded patients were those unable to take care of themselves, who had severe or critical COVID-19, or were older than 65 years. After admission, patients would receive empirical antiviral treatment and supportive care followed by PCR testing and a chest CT scan according to the standard protocol.

In Feb 2020, 16 temporary cabin hospitals were built in a short time in different districts of Wuhan, China, the epicenter of COVID-19 outbreak, to relieve the strain on the regular health care system. Asymptomatic or mildly symptomatic adult patients were admitted to the geographically closest temporary cabin hospital. Before being admitted to the cabin hospital, all patients were diagnosed as having COVID-19 following laboratory confirmation of SARS-CoV-2 infection using polymerase chain reaction (PCR) testing. In addition, every patient underwent an initial chest CT scan at admission. Patients who could not take care of themselves, had severe or critical COVID-19, or were older than 65 years were excluded (Figure 1). Diagnostic criteria for severe and critical patients were in accordance with the Guidelines for COVID-19 Diagnosis and Treatment (Trial version 5) published by the National Health Commission of the People's Republic of China (National Health Commission of China, 2020). COVID-19 patients meeting any of the following criteria were diagnosed as severe: (1) respiratory distress, respiratory rate ≥30 times/min; (2) oxygen saturation ≤ 93% at rest; and (3) PaO_2_/FiO_2_ ≤ 300 mmHg. COVID-19 patients with any of the following criteria were diagnosed as critical: (1) respiratory failure and mechanical ventilation needed, (2) shock, or (3) organ failure and intensive care unit (ICU) admission needed for monitoring and treatment. After admission, the patients would receive empirical antiviral treatment and supportive care followed by consecutive PCR testing and chest CT scan according to a standard protocol (Figure 1). In this study, we collected data retrospectively from Wuhan Sports School cabin hospital, which was overseen by the Maternal and Child Health Hospital of Hubei Province, Wuhan, China. This study was approved by the Medical Ethical Committee of the Maternal and Child Health Hospital of Hubei Province [IEC (LW 024), 2020]. Written informed consent was obtained from each enrolled patient.

### Data Collection

The Wuhan Sports School cabin hospital started admitting patients on Feb 22, 2020 and was closed on Mar 8, 2020. In total, 265 patients were admitted to the cabin hospital. Thirteen patients were excluded from the analysis due to the lack of reliable medical records. After admission, all patients were subjected to nucleic acid testing at least twice and chest CT scans at least once. Ninety patients underwent a complete blood count test. The demographic data, clinical records, and laboratory findings were reviewed by two physicians (YL and CC).

Before and after admission, all patients' throat swab samples were collected and sent to the Hubei Provincial Center for Disease Control and Prevention to be tested for SARS-CoV-2 following the WHO guidelines. A positive test result confirmed the diagnosis of COVID-19. For patients who tested negative for the first time after admission, a second test was arranged within the next 24–48 h (Figure 1). Patients who had a negative result twice in a row in the nucleic acid test were considered virus-free. The duration of virus clearance was defined as the time from the first positive test to the first negative test.

All chest CT scans were reviewed by two radiologists (XD and WS). After admission, patients who tested negative twice on consecutive nucleic acid tests were subjected to at least one chest CT scan. Patients who did not present with a pneumonia-like lesion on the first chest CT scans after admission were not subjected to subsequent follow-up chest CT scans. Therefore, data of follow-up chest CT scans were not available for those patients. The evolution of lesions on follow-up CT was rated as no significant change, improvement, resolution, or aggravation, after comparison with the patient's previous chest CT scans. Decisions were made by consensus.

Patients would be discharged from the cabin hospital if they met all of the following criteria: nucleic acid test negative twice in a row; chest CT showed no aggravation; improved symptoms; and normal body temperature for three consecutive days. After discharge from the cabin hospital, patients were transferred to a designated place to be quarantined for 14 days. Patients would be referred to a designated hospital for more intensive care if they showed signs of disease progression, [i.e., chest CT showed aggravation or met the diagnostic criteria for severe or critical COVID-19 according to the Guidelines for COVID-19 Diagnosis and Treatment (Trial version 5) (National Health Commission of China, 2020) (Figure 1)].

### Statistical Analysis

Summary statistics for categorical variables were presented as number (percentage), for continuous variables were presented as mean (range). The association between two categorical variables was tested with Fisher's exact test or Pearson chi-square test. The association of blood test panel and patient symptomatic status was assessed by one-way ANOVA. All tests were 2-sided with *P* < 0.05 as significance threshold. The analysis was performed with SAS 9.4 unless otherwise specified.

## Results

The Wuhan Sports School cabin hospital admitted a total of 265 COVID-19 patients. Thirteen patients were excluded from the analysis due to missing medical records. The remaining 252 patients were included in the final analysis.

**Figure 2:**
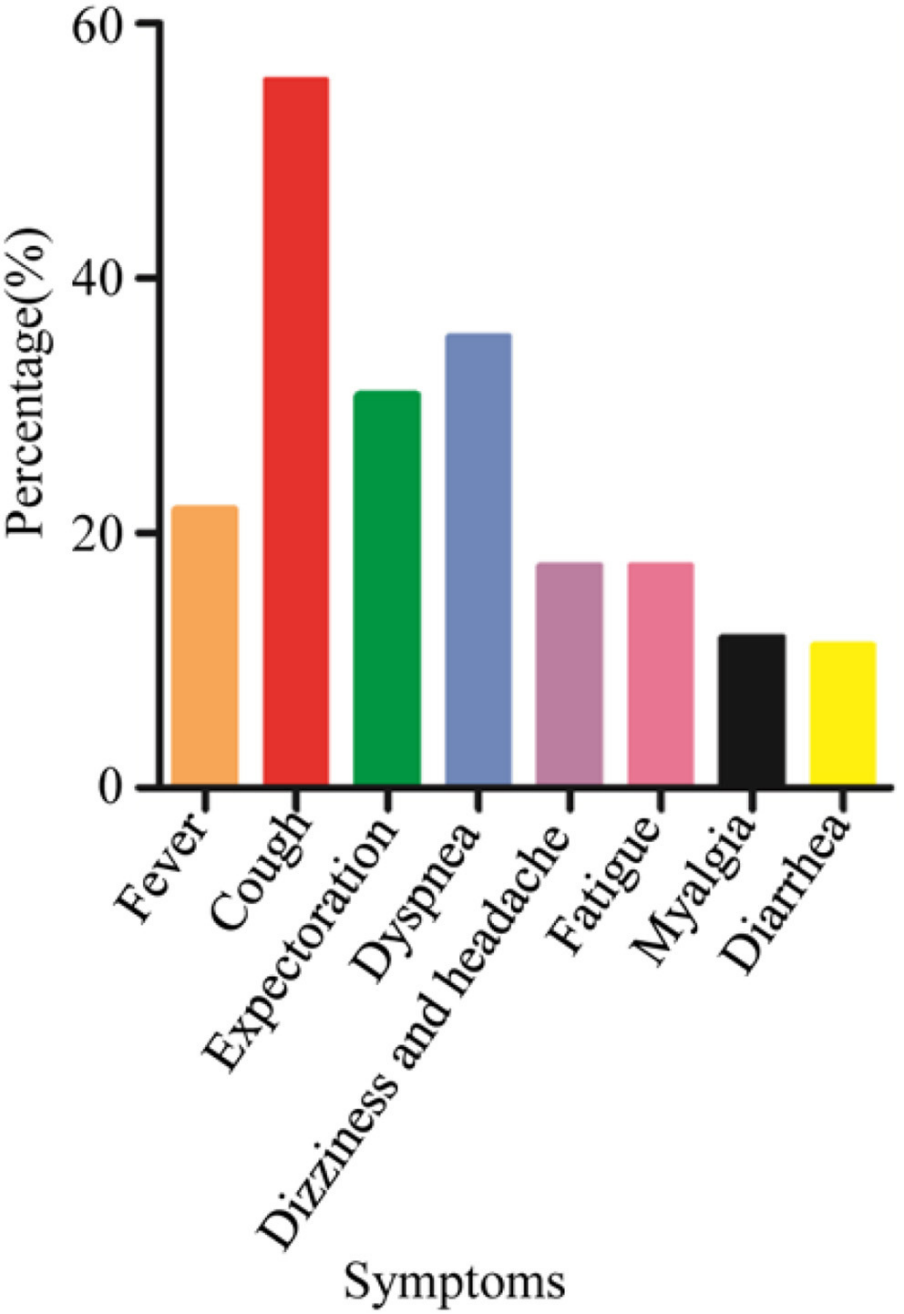
Symptoms of non-severe coronavirus disease 2019 patients at a cabin hospital. The most common symptom was coughing. Other common symptoms included dyspnea, expectoration, and fever. Less common symptoms included dizziness and headache, fatigue, myalgia, and diarrhea.

All patients had laboratory-confirmed SARS-CoV-2 infection; 138 (54.76%) had more than two family members who developed COVID-19. The probability of family clustering was similar between asymptomatic and symptomatic patients (59.70 vs. 61.64%, *P* = 0.79). One hundred and twenty-five (49.6%) patients were male and 127 (50.4%) were female; 74 (29.4%) patients were asymptomatic and 178 (70.6%) were symptomatic. The median age of the patients was 46 years (range, 8–65 years); 31 (12.3%) patients were aged 60 years or older and four (1.9%) were younger than 20. Older patients were more likely to be symptomatic than younger patients (*P* = 0.05). Among the 178 patients who showed symptoms, the most common symptom was coughing (55.6%). The other common symptoms were dyspnea (35.4%), expectoration (30.9%), and fever (21.9%). Less common symptoms were dizziness and headache (17.42%), fatigue (17.42%), myalgia (11.8%), and diarrhea (11.2%) (Figure 2). Forty (15.9%) patients had at least one comorbidity. Hypertension was the most common comorbidity (7.5%) and diabetes was the second most common one (4.8%).

Ninety patients underwent a complete blood count test. The majority of patients had a normal counts of white blood cells, lymphocytes, neutrophils, and platelets, and normal hemoglobin. Whereas, the lymphocyte count was within normal range for all patients, the symptomatic patients had a significantly higher lymphocyte count than asymptomatic patients (*P* = 0.03).

Among all the 252 patients, 119 (47.2%) were found to have bilateral pneumonia and 52 (20.6%) had unilateral pneumonia on the initial chest CT scan. Eighty-one (32.1%) patients had no signs of pneumonia on the initial and first CT scan; a subsequent chest CT was not ordered for these patients. On the follow-up chest CT scan, 8/119 (6.7%) bilateral pneumonia patients showed signs of aggravation whereas none of the unilateral patients showed any signs of aggravation. The bilateral pneumonia patients had a significantly higher chance of having disease aggravation than the unilateral pneumonia patients (*P* = 0.02). Symptomatic patients were more likely to have bilateral pneumonia (*P* < 0.0001) and less likely to show improvement of pneumonia than asymptomatic patients (*P* = 0.0002). Among all 252 patients, 221 (87.7%) tested negative twice in a row within an average of 7.9 days (range, 3–39 days). Twenty-three patients (9.1%) had negative test results within 5 days. The probability of being virus-free was similar between asymptomatic and symptomatic patients (93.15 vs. 86.44%, *P* = 0.13). However, non-symptomatic patients were more likely to have a negative test result within 5 days than symptomatic patients (17.81 vs. 5.65%, *P* = 0.003) (Table 1).

**Table 1:** Patient characteristics table for 252 patients who were admitted to the temporary cabin hospital between Feb 22, 2020 and Mar 8, 2020.

	**Asymptomatic (*n* = 74)**	**Symptomatic (*n* = 178)**	***p*-value^**#**^**
Gender			0.64
Male	35 (47.30)	90 (50.56)	
Female	39 (52.70)	88 (49.44)	
Age (years)			0.05
<10	1 (1.35)	0	
10–19	3 (4.05)	0	
20–29	14 (18.92)	22 (12.36)	
30–39	17 (22.97)	37 (20.79)	
40–49	15 (20.27)	47 (26.40)	
50–59	16 (21.62)	49 (27.53)	
≥60	8 (10.81)	23 (12.92)	
Chest CT diagnosis			<0.0001
No pneumonia	38 (51.35)	43 (24.16)	
Unilateral pneumonia	15 (20.27)	37 (20.79)	
Bilateral pneumonia	21 (28.38)	98 (55.06)	
Chest CT follow-up			0.0002
No change	8 (10.81)	40 (22.47)	
Improvement	14 (18.92)	65 (36.52)	
Resolution	8 (10.81)	15 (8.43)	
Aggravation	1 (1.35)	7 (3.93)	
Not available	43 (58.11)	51 (28.65)	
Virus clear^*^			0.13
No	5 (6.85)	24 (13.56)	
Yes	68 (93.15)	153 (86.44)	
Virus clear within 5 days^*^			0.003
No	60 (82.19)	167 (94.35)	
Yes	13 (17.81)	10 (5.65)	
Comorbidities			0.07
None	67 (90.54)	145 (81.46)	
With one or more	7 (9.46)	33 (18.54)	
Blood test panel^**^			
White blood cell count	5.03 (4.00–6.66)	5.12 (4.00–7.51)	0.66
Hemoglobin (g/L)	140.10 (118.00–165.00)	138.10 (89.00–169.00)	0.62
Platelet count	221.38 (167.00–342.00)	228.17 (114.00–430.00)	0.64
Neutrophil count	2.78 (1.83–3.77)	2.76 (1.80–4.05)	0.87
Lymphocyte count	1.58 (0.21–2.69)	1.78 (0.90–2.62)	0.03
Monocyte count	0.44 (0.11–0.70)	0.40 (0.10–1.28)	0.41
Family cluster			0.79
No	27 (40.30)	61 (38.36)	
Yes	40 (59.70)	98 (61.64)	

The summary values were presented as number (percentage) for categorical variables, mean (range) for continuous variables.

# P-value based on chi-square test or fisher's exact test for categorical variables, as appropriate, and one-way ANOVA test for continuous variables.

* Virus clear was defined as test negative twice in a row. Only initially nucleic acid test positive patients were calculated.

** *The blood test panel result was summarized from 21 asymptomatic patients and 69 symptomatic patients, due to data availability*.

In total, 183 (72.6%) patients were discharged when the temporary cabin hospital was closed. Sixty-nine (27.4%) patients were referred to the designated hospital. Thirty-six (14.3%) patients were referred because the cabin hospital was going to be closed, which was the most common reason for referral. Only 23 (9.1%) patients were referred due to progression of pneumonia. The patient's referral status was not significantly associated with age or sex (*P* = 0.46 and *P* = 0.95, respectively). Patients with symptoms were more likely to be referred to a designated hospital (*P* = 0.004). Patients with aggravation of pneumonia on follow-up chest CT were more likely to be referred (*P* < 0.0001). Patients who did not achieve a virus-free status were also more likely to be referred (*P* < 0.0001). The referral status was not associated with whether the patients achieved a virus-free status within 5 days (*P* = 0.86) (Table 2).

**Table 2:** Comparison of clinical features of referred patients vs. non-referred patients.

	**No referral (*n* =183)**	**Referred to a designated hospital (*n* = 69)**	***p*-value^**#**^**
Gender			0.95
Male	91 (49.73)	34 (49.28)	
Female	92 (50.27)	35 (50.72)	
Age (years)			0.46
<10	1 (0.55)	0	
10–19	3 (1.64)	0	
20–29	29 (15.85)	7 (10.14)	
30–39	43 (23.50)	11 (15.94)	
40–49	43 (23.50)	19 (27.54)	
50–59	43 (23.50)	22 (31.88)	
≥60	21 (11.48)	10 (14.49)	
Chest CT findings			<0.0001
No pneumonia	75 (40.98)	6 (8.70)	
Unilateral pneumonia	40 (21.86)	12 (17.39)	
Bilateral pneumonia	68 (37.16)	51 (73.91)	
Chest CT follow-up			<0.0001
No change	17 (9.29)	31 (44.93)	
Improvement	63 (34.43)	16 (23.19)	
Resolution	22 (12.02)	1 (1.45)	
Aggravation	2 (1.09)	6 (8.70)	
Not available	79 (43.17)	15 (21.74)	
Virus clear^*^			<0.0001
No	0	29 (42.03)	
Yes	181 (100)	40 (57.97)	
Virus clear within 5 days^*^			0.86
No	164 (90.61)	63 (91.30)	
Yes	17 (9.39)	6 (8.70)	
Comorbidities			0.02
None	160 (87.43)	52 (75.36)	
With one or more	23 (12.57)	17 (24.64)	
Symptomatic			0.004
No	63 (34.43)	11 (15.94)	
Yes	120 (65.57)	58 (84.06)	

The summary values were presented as number (percentage) for categorical variables, mean (range) for continuous variables.

# P-value based on chi-square test or fisher's exact test for categorical variables, as appropriate, and one-way ANOVA test for continuous variables.

* *Virus clear was defined as tested negative twice in a row. Only initially nucleotide test positive patients were calculated*.

## Discussion

To our knowledge, this report is the first retrospective single center study of COVID-19 in a Chinese temporary cabin hospital comparing asymptomatic and symptomatic patients.

This study included 252 COVID-19 patients with or without mild symptoms. Seventy-four (29.4%) patients had no symptoms, consistent with a study that observed 34.6% of COVID-19 patients were asymptomatic on-board the Princess Cruise (Mizumoto et al., 2020). Thirty-eight asymptomatic patients were found to not have pneumonia on chest CT scan. Thirty-six asymptomatic patients were found to have pneumonia, which is consistent with a previous report that some asymptomatic patients also had abnormal CT imaging features (Shi et al., 2020). Among the 178 patients with mild symptoms, cough was the most common symptom (55.6%), which is consistent with a previous study on mild symptomatic pneumonia (Chen H. et al., 2020). This finding is different from what was reported in severe symptomatic patients, where fever was found to be the most common symptom (Chen N. et al., 2020; Zhou et al., 2020a). We observed bilateral pneumonia patients in 47.2% of patients in our study, whereas Chen and colleagues observed bilateral pneumonia in 75% of patients who had severe pneumonia (Chen N. et al., 2020). We speculate that severely symptomatic patients tend to have severe inflammation in their lungs, which more commonly leads to fever. We found that patients were more likely to be non-symptomatic if they met one or more of the following criteria: young age, unilateral pneumonia, and achieved virus-free status within 5 days.

Previous studies reported that the median duration to viral shedding was 19.0 and 24.0 days in patients with severe disease and critical disease status (Zhou et al., 2020a). Our study showed that the median duration to viral shedding was 7 days for both asymptomatic and mild symptomatic patients. Twenty-three (9.1%) patients reach a virus-free status within 5 days. It can be inferred that patients without severe disease status could clear the virus in a shorter time frame. We revealed that asymptomatic patients were more likely to clear the virus within 5 days than patients with mild symptoms (17.8 vs. 5.6%, *P* = 0.003). However, the average duration of viral shedding was not significantly different between the two groups (mean time in days, 7.90 vs. 7.88, *P* = 0.97). This suggests that asymptomatic and mild symptomatic patients carry the virus and are infectious for a similar amount of time. In addition, a recent study found that the viral load detected in asymptomatic and symptomatic patients was similar (Zou et al., 2020). We also found that the probability for family clustering is similar between asymptomatic and symptomatic patients. These results suggest that non-severe COVID-19 patients can transmit the disease regardless of their symptomatic status. Therefore, if asymptomatic patients are not identified in a timely manner and quarantined, they could become moving sources of infection and lead to massive transmission of disease (Gostic et al., 2020; Zhou et al., 2020b). It is highly recommended that such asymptomatic patients be identified and quarantined to eliminate the transmission of SARS-CoV-2.

Umifenovir hydrochloride capsules (Arbidol), an anti-viral drug, and Chinese herbs were given to every patient admitted to the cabin hospital. Oxygen therapy and antipyretics were also given if needed. When the cabin hospital was about to be closed, 183 (72.6%) patients were discharged because they met the following criteria: achieved a virus-free status as indicated by testing negative twice in a row in the nucleic acid test, showed no aggravation on the chest CT scan, and had an improvement in symptoms. Consistent with the meta-analysis by Yang et al. (2020), we found that hypertension and diabetes were the two most common comorbidities in patients with COVID-19. Older patients and those with comorbidities were more likely to experience disease aggravation and require referral to the regular hospital for more intensive care, probably because they had weaker immune systems (Wang et al., 2020b; Wu et al., 2020). Among the 69 patients who were referred to a designated hospital, only 23 of them were due to worsened disease, which constituted <10% of the total number of patients. If medical caregivers monitor those at higher risk of disease progression, COVID-19 patients can be safely quarantined.

In conclusion, non-severe COVID-19 patients can transmit the disease regardless of their symptomatic status. More attention should be given to identifying and quarantining asymptomatic patients to eliminate the transmission of SARS-CoV-2. If both asymptomatic and symptomatic non-severe COVID-19 patients can be appropriately quarantined, community and family-clustered transmission may be greatly reduced (Bai et al., 2020; Chan et al., 2020).

A limitation of this study is that all patients were administered the same treatment. There was no control arm so we cannot reach any conclusions regarding the effects of medication. The other limitation is that follow-up after patient discharge has not yet been completed. Further study is needed to evaluate the long-term effects of COVID-19 in patients.

## Data Availability Statement

All datasets presented in this study are included in the article/supplementary material.

## Ethics Statement

The studies involving human participants were reviewed and approved by Medical Ethical Committee of the Maternal and Child Health Hospital of Hubei Province, Tongji Medical College, Huazhong University of Science and Technology (Wuhan, Hubei, China). Written informed consent to participate in this study was provided by the participants' legal guardian/next of kin. All patients in the cabin hospital signed informed consent form for the publication of their clinical details.

## Author Contributions

YL and CW had full access to all the data in the study and take responsibility for the integrity of the data and the accuracy of the data analysis. Concept and design done by XY, HG, and CW. Acquisition, analysis, or interpretation of data done by JS, JX, JD, and LC. Drafting of the manuscript done by QM and XW. Statistical analysis done by YL, YY, and LG. Administrative, technical, or material support done by XY, WL, and XY. Supervision done by HG. All authors read and approved the final manuscript.

## Conflict of Interest

The authors declare that the research was conducted in the absence of any commercial or financial relationships that could be construed as a potential conflict of interest.
